# Stimulating human prefrontal cortex increases reward learning

**DOI:** 10.1016/j.neuroimage.2023.120029

**Published:** 2023-03-15

**Authors:** Margot Juliëtte Overman, Verena Sarrazin, Michael Browning, Jacinta O’Shea

**Affiliations:** a Wellcome Centre for Integrative Neuroimaging, University of Oxford, OX3 9DU; b Department of Psychiatry, Warneford Hospital, University of Oxford, OX3 7JX; c Oxford Centre for Human Brain Activity (OHBA), University of Oxford, OX3 7JX

**Keywords:** Prefrontal tDCS, affective bias, reinforcement learning, learning rate, brain stimulation

## Abstract

Work in computational psychiatry suggests that mood disorders may stem from aberrant reinforcement learning processes. Specifically, it has been proposed that depressed individuals believe that negative events are more informative than positive events, resulting in higher learning rates from negative outcomes (Pulcu & Browning, 2019). In this proof-of-concept study, we investigated whether transcranial direct current stimulation (tDCS) applied to dorsolateral prefrontal cortex, as commonly used in depression treatment trials, might change learning rates for affective outcomes. Healthy adults completed an established reinforcement learning task (Pulcu & Browning, 2017) in which the information content of reward and loss outcomes was manipulated by varying the volatility of stimulus-outcome associations. Learning rates on the tasks were quantified using computational models. Stimulation over dorsolateral prefrontal cortex (DLPFC) but not motor cortex (M1) increased learning rates specifically for reward outcomes. The effects of prefrontal tDCS were cognitive state-dependent: tDCS applied *during* task performance increased learning rates for wins; tDCS applied *before* task performance decreased both win and loss learning rates. A replication study confirmed the key finding that tDCS to DLPFC during task performance increased learning rates specifically for rewards. Taken together, these findings demonstrate the potential of tDCS for modulating computational parameters of reinforcement learning that are relevant to mood disorders.

## 1 Introduction

The ability to learn from experience is central to adaptive decision making. Bayesian accounts of reinforcement learning posit that optimal learners should determine which events are most *informative* (i.e., most useful for predicting future outcomes) and weight these accordingly when making choices (Behrens, Woolrich, Walton, & Rushworth, 2007; Browning, Behrens, Jocham, O’Reilly, & Bishop, 2015; MacKay, 2003; Nassar et al., 2012). The information content of an event depends in part on the volatility of the association being learned. When action-outcome contingencies change frequently, each new observation is relatively more informative about the current state of the association than more distant outcomes. Predictions should therefore be updated more rapidly for volatile than stable associations, i.e. to optimise choice behaviour individuals should show higher learning rates in volatile than stable environments (Pulcu & Browning, 2019). In keeping with this theory, healthy adults flexibly adapt their learning rates to match the volatility (or information content) of events (Behrens et al., 2007; Browning et al., 2015; Nassar et al., 2012). The learning rate can therefore be interpreted as a measure that reflects individuals’ estimate of the information content of events. Moreover, humans can maintain separate estimates of the information content of positive and negative events (Pulcu & Browning, 2017).

Since the optimal learning rate depends on the informativeness of outcomes, individuals need to adjust their behaviour flexibly to the statistics of rewards and punishments (Behrens et al., 2007; Browning et al., 2015; Nassar et al., 2012). An emerging line of computational psychiatry research suggests that aberrant tracking of these statistical properties could form a core mechanism underpinning affective disorders (Browning et al., 2015; Gagne, Zika, Dayan, & Bishop, 2020; Pulcu & Browning, 2019). Depression is typically characterised by a negative cognitive bias, i.e. attention, information processing and memory are biased towards negative rather than positive information (Eshel & Roiser, 2010; Mathews & MacLeod, 2005). Negative biases are hypothesised to causally lead to depressive symptoms (Disner, Beevers, Haigh, & Beck, 2011), and a reduction in negative biases is hypothesised to be one mechanism of effective antidepressant treatments (Godlewska, Browning, Norbury, Cowen, & Harmer, 2016). Building on preclinical work, it has been proposed that negative biases in depression may stem from a tendency to overestimate the information content of negative relative to positive events (Pulcu & Browning, 2019). In a reinforcement learning model, this would manifest in increased punishment relative to reward learning rates. Increased punishment learning rates (Aylward et al., 2019; Pike & Robinson, 2022) and decreased reward learning rates (Brown et al., 2021) have both been observed in depression. This could in turn lead to a negative cognitive bias. If an individual considers negative outcomes to be more informative for predicting future outcomes than positive outcomes, their attention will be biased towards negative (rather than positive) outcomes. On a behavioural level, they might change their behaviour very quickly in response to negative outcomes (i.e. increased punishment learning rate). As an illustrative example, a belief that negative events are highly informative may cause an individual to pay more attention to criticism than praise at work. As well as impairing mood and self-confidence, this belief likely increases the influence of negative feedback on a person’s decision whether to pursue a potentially fruitful project (i.e. increased punishment learning rate leads to quick changes of behaviour in response to negative feedback). From this perspective, a potential therapeutic approach to counter-act negative biases that causally maintain depression would be to re-balance reward and punishment learning rates so that positive feedback is taken into account to the same extent as negative feedback when deciding whether to change behaviour or not.

In this proof-of-concept study, we tested whether transcranial direct current stimulation applied to dorsolateral prefrontal cortex (DLPFC) might increase reward vs. punishment learning rates in healthy adults. Depression is consistently linked with hypoactivity of the left dorsolateral prefrontal cortex (DLPFC) and concurrent hyperactivity of the right DLPFC (Disner et al., 2011; Grimm et al., 2008; Koenigs & Grafman, 2009). DLPFC forms part of distributed reward learning circuitry (Haber & Knutson, 2010; Lee, Seo, & Jung, 2012) implicated in tracking the volatility of reward outcomes (Farashahi, Donahue, Hayden, Lee, & Soltani, 2019; Massi, Donahue, & Lee, 2018). Hence, modulating DLPFC could influence both neural and cognitive mechanisms underlying depression. In clinical trials, transcranial direct current stimulation (tDCS) of DLPFC applied at rest improves depression (Brunoni et al., 2016; Razza et al., 2020; Shiozawa et al., 2014). One of the most commonly applied montages is a “bifrontal” montage with the anode placed over the left, and the cathode over the right DLPFC. Since anodal tDCS and cathodal tDCS have been shown to have excitatory and inhibitory effects on (motor) cortical excitability, respectively (Bergmann et al., 2009; Michael A Nitsche & Paulus, 2000), this montage has been used in depression with the aim to re-balance the physiological asymmetry commonly observed between left and right DLPFC (Disner et al., 2011; Grimm et al., 2008; Koenigs & Grafman, 2009). We reasoned that the functional impact (and consequent therapeutic potential) of bifrontal tDCS could be enhanced by stimulating during a reinforcement learning task. Our aim was to exploit tDCS-induced neuroplasticity to target and change reinforcement learning processes thought to causally maintain depression. Since negative biases are hypothesised to stem from increased punishment learning (Aylward et al., 2019; Eshel & Roiser, 2010; Pike & Robinson, 2022) we were particularly interested in testing whether tDCS can selectively increase reward learning rates or decrease punishment learning rates, both of which might be beneficial to counter-act negative biases.

Here we performed a proof-of-concept test of this hypothesis via a series of experiments in healthy adults. We applied tDCS to bilateral DLPFC, a montage typically used in clinical treatment trials with the aim to restore the normal cortical excitability balance between left and right DLPFC that is commonly altered in depression. We predicted that, compared to sham stimulation, tDCS over DLPFC would lead to a relative increase of reward vs. punishment learning rates, i.e. an increase in reward and/or decrease in punishment learning. Since tDCS enhances activity-dependent synaptic plasticity (Fritsch et al., 2010; O’Shea et al., 2017), this effect should be cognitive-state dependent. That is, we predicted increased reward vs. punishment learning rates specifically with tDCS applied *during* but not before learning. Finally, we predicted the effect would be anatomically specific, with stimulation of prefrontal but not motor cortex (M1) inducing a relative increase in reward learning rates.

One statistical characteristic that has been shown to influence learning rates is the volatility of action-outcome associations (Behrens et al., 2007; Browning et al., 2015), and depression is associated with difficulties in adjusting learning rates to volatility (Browning et al., 2015; Gagne et al., 2020). We therefore used an established reinforcement learning paradigm in which the volatility of rewards and punishments is manipulated independently across blocks (Browning et al., 2015; Pulcu & Browning, 2017; Pulcu et al., 2019), enabling us to quantify reward and punishment learning rates separately. We used a single dose of the tDCS montage and protocol commonly used in depression treatment trials, applying stimulation bilaterally over DLPFC.

## 2 Materials and Methods

### 2.1 Participants

Eighty healthy adults (45 female, mean age = 24.71, SD ± 5.08) were recruited from the community via local advertisements for four independent studies. Exclusion criteria were left-handedness, a history of psychiatric disorders, neurological illness, use of psychoactive medication, personal or family history of epileptic fits or seizures, and any other contraindications to tDCS. The experimental protocol was approved by the University of Oxford Central University Ethics Committee (RE48995/RE002) and all participants gave written informed consent prior to the study. Demographic details of the participants are provided in Table 1.

### 2.2 Study overview and experimental design

Participants took part in one of four stimulation studies, each consisting of two tDCS and reward learning sessions. 20 participants were recruited for each study. An a priori power analysis was not possible since no prior evidence on valence-specific effects of tDCS on learning rates was available which could have provided an effect size estimate. The sample size was therefore chosen pragmatically based on resource constraints (see Discussion for sensitivity power analysis and further comments). All participants underwent both active and sham tDCS sessions in a cross-over, double-blind design. Stimulation order was counterbalanced in all groups and sessions were scheduled at least one week apart to minimise carryover effects of repeated learning and/or tDCS. In Study 1, tDCS was applied to DLPFC during task performance (“tDCS during task”) to test for the predicted increase in reward learning. In Study 2, tDCS was applied to DLPFC prior to task performance (“tDCS before task”) to determine the cognitive state dependence of any DLPFC stimulation effect. In Study 3, tDCS was delivered over primary motor cortex (M1) during task performance to determine the anatomical specificity of tDCS effects during task performance. Study 4 aimed to replicate the findings of Study 1, to evaluate the consistency of behavioural changes induced by prefrontal tDCS applied during task performance.

### 2.3 Questionnaires

Symptoms of depression and anxiety (see Table 1) were assessed at baseline with the Beck Depression Inventory-II (BDI) (Beck, Steer, & Brown, 1996) and the Trait subscale of the State-Trait Anxiety Inventory (STAI-Trait) (Spielberger, Gorsuch, Lushene, Vagg, & Jacobs, 1983). Higher scores on these tasks indicate greater symptoms of depression and anxiety, respectively. For the BDI, a cut-off score of 13 was suggested for clinically relevant symptoms of depression (Beck et al., 1996). The STAI-Trait was not designed for clinical diagnosis and therefore has no formal cut-off points, although scores in the 40-80 range are commonly interpreted as clinically significant levels of anxiety. As expected, participants tended to score in the low range on both the BDI and STAI-Trait (see Table 1). To monitor potential changes in acute mood and anxiety across the tDCS sessions, participants completed the Positive and Negative Affect Scales (PANAS) (Watson, Clark, & Tellegen, 1988) and the State-Trait Anxiety Inventory (STAI-State) (Spielberger et al., 1983) immediately before and after completion of the task (see Information Bias Learning Task (IBLT) below). All scores and analyses of the PANAS and STAI-State are reported in the Extended Data Table S2.

### 2.4 Information Bias Learning Task (IBLT)

The Information Bias Learning Task (IBLT) is a computerised reinforcement learning paradigm which has been described in detail previously (Pulcu & Browning, 2017; Pulcu et al., 2019). The IBLT was presented on a laptop computer using Presentation® software (Neurobehavioral Systems, Inc., Berkeley, CA, www.neurobs.com). On every trial, a fixation cross in the centre of the screen was flanked by two abstract shapes (letters selected from the Agathodaimon font). Participants were asked to choose the shape they believed would result in the best outcome via a button press, after which a win (+10p) and a loss (-10p) outcome appeared in randomised order above or below the shapes. Participants’ accumulated total winnings were displayed under the fixation cross and updated at the beginning of the subsequent trial. The win and loss outcomes were independent, such that on any trial a specific shape could be associated with one, both, or neither of the outcomes. Participants therefore had to form separate predictions of the likelihood of the win and the loss appearing over a specific shape, and select the optimal choice based on those estimates.

**Figure 1:**
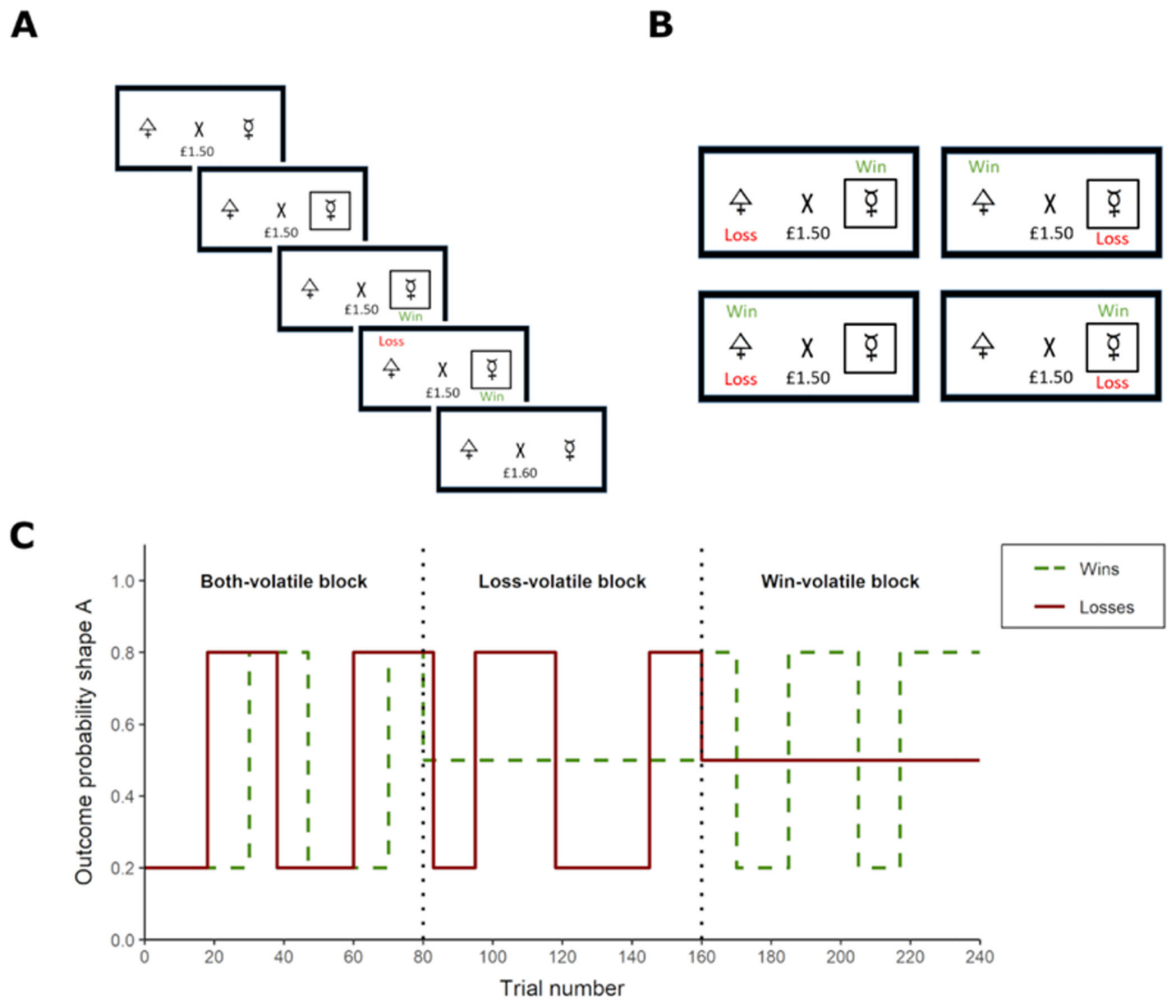
**(A)** Schematic representation of a trial on the Information Bias Learning Task (IBLT; Pulcu & Browning, 2017). After showing the fixation cross and total amount of money won, two abstract shapes are presented on either side of the cross. Once the participant has chosen one of the shapes via a button press, a black frame appears around that shape and a win and loss outcome appear successively in randomised order. A win outcome leads to an increase of 10p, whereas a loss outcome represents a decrease of 10p from the total amount of money won. The total amount of money is updated at the start of the next trial. The aim of the task is to maximise earnings by learning the probabilities of the win and the loss appearing over the respective shapes. **(B)** The four possible outcomes on a task trial. The win and the loss outcomes are independently associated with one of the shapes, allowing for a shape to be associated with one, both, or neither of the outcomes at a given time. **(C)** Volatility of the win (green) and loss (red) outcomes across blocks of the Information Bias Learning Task (IBLT). Volatility for the two outcomes is manipulated independently across task blocks, either switching between 20% and 80% choice-outcome associations or remaining stable at 50% choice-outcome associations. If both wins and losses are volatile, participants should rapidly update their predictions for both types of outcomes (i.e., have a high learning rate). In the ‘Win-volatile’ blocks participants should adopt a high learning rate for wins and a low learning rate for losses, whereas the opposite approach should be taken in the ‘Loss-volatile’ blocks.

**Figure 2:**
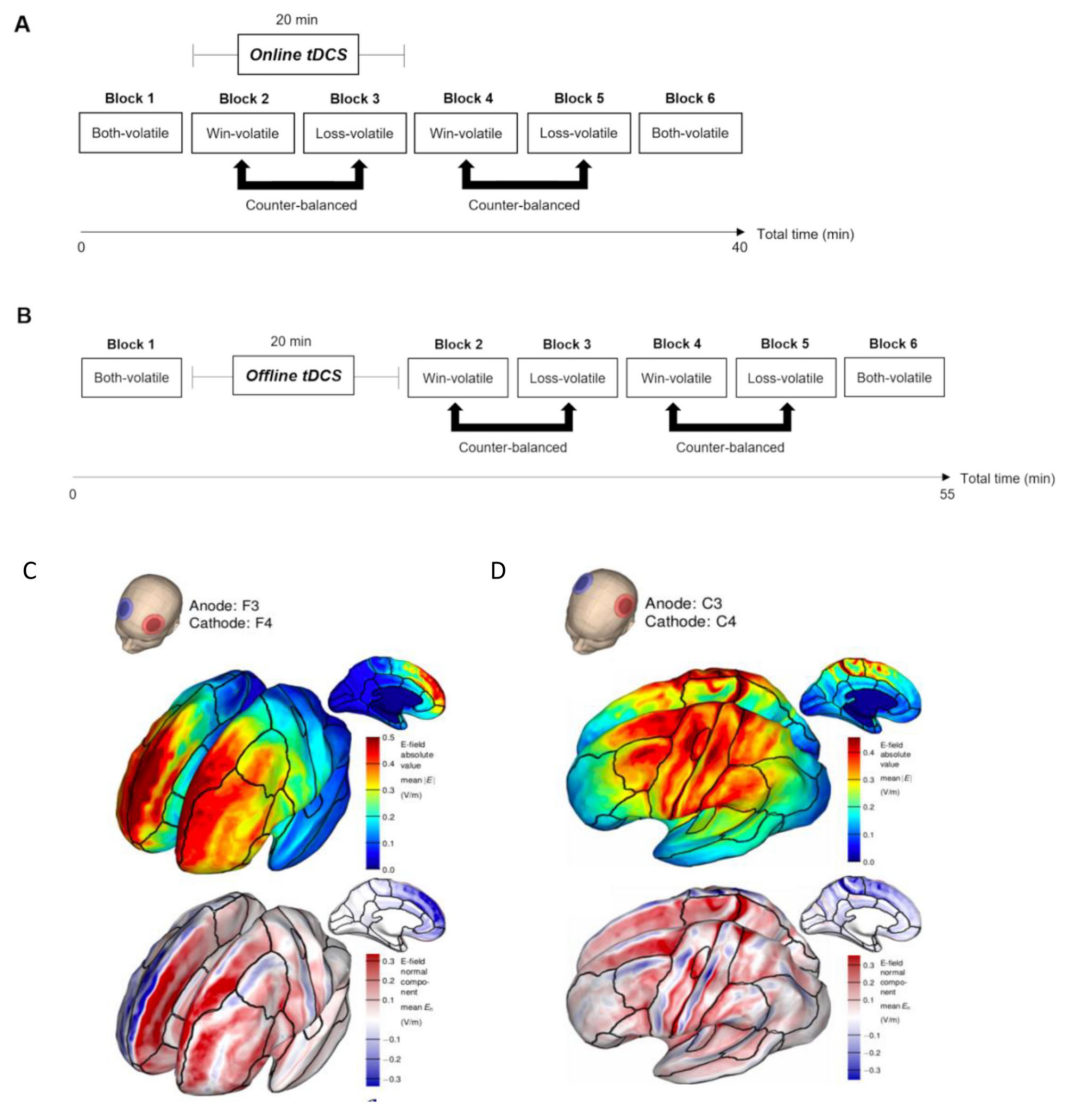
**(A)** Structure and timeline of the tDCS paradigm combined with tDCS applied during task performance (Study 1, 3, and 4). Participants completed six blocks of the Information Bias Learning Task (IBLT). The task started and ended with a ‘Both-volatile’ block in which win and loss outcomes are equally informative. The participants were then presented with two ‘Win-volatile’ and two ‘Loss-volatile’ blocks, with block type order counterbalanced across participants. tDCS was applied during Blocks 2-3 of the IBLT. **(B)** Study 2 structure and timeline, in which DLPFC tDCS was applied prior to task performance. **(C)** Simulation of the electric field (top) and normal component (bottom) induced in the brain by the bilateral prefrontal tDCS montage, with the anodal electrode (red) over the left DLPFC (F3) and the cathode (blue) over the right DLPFC (F4). **(D)** Simulation of the electric field induced in the brain by the bilateral motor cortex tDCS montage, with the anodal electrode (red) over left M1 and the cathode (blue) over right M1. While the electric field strength induced in the two hemispheres is similar, the left hemisphere primarily receives anodal (red), and the right hemisphere cathodal stimulation (blue)(normal component). (C) and (D) are adapted from (Laakso et al., 2016) with permission.

Participants completed six task blocks of 80 trials each, with a fixed 30-second rest period between blocks. The same two shapes were used within a task block, and different shapes were used across task blocks. The volatility of shape-outcome contingencies was varied across task blocks to manipulate the relative information content of the win and loss outcomes (see Figure 1). During volatile (i.e., informative) task periods, the association of an outcome with shape ‘A’ reversed between 20% and 80% every 14 to 30 trials. During stable (i.e., uninformative) task periods, the association of an outcome with shape ‘A’ remained constant at 50%. The probability of an outcome appearing over shape ‘B’ was calculated as 1 – shape ‘A’. In blocks 1 and 6, both the win and loss outcome associations were volatile. The aim of these ‘Both-volatile’ blocks was to measure the extent to which participants preferentially learned from equally informative positive and negative outcomes. In blocks 2-5, one outcome was volatile whereas the other remained stable. These blocks, in which only win or only loss outcomes were volatile, will be referred to as ‘Win-volatile’ and ‘Loss-volatile’ blocks, respectively. The Win- and Loss-volatile blocks enabled us to simultaneously test for potential specific effects of tDCS on learning for outcomes of different valences (positive vs. negative) and levels of information content (informative vs. uninformative). Win- and Loss-volatile blocks alternated, with block order (i.e. Win-volatile or Loss-volatile block first) counterbalanced across participants in each study (see Figure 2.A).

In all tDCS studies, block 1 (‘Both-volatile’) was completed prior to stimulation to provide a baseline measure of learning rates for win and loss outcomes. In the “tDCS during task” studies (Study 1, 3, and 4), stimulation was applied *during* task blocks 2 and 3 (20 minutes). Immediately after tDCS offset, participants completed blocks 4-5, to test if effects of tDCS applied during task performance persisted post stimulation (see Figure 2.A). Block 6 (‘Both-volatile’) was used to measure potential changes in learning behaviour by the end of the task compared to Block 1 in case the effects of tDCS last until the end of task performance. In the ‘tDCS before task’ study (Study 2), tDCS was applied first, while participants sat at rest, followed by task blocks 2-6 (see Figure 2.B).

The goal of the study was to test if prefrontal stimulation during the task specifically increased relative learning rates for positive vs. negative outcomes. This would manifest as a relative increase in learning rates for wins vs. losses.

### 2.5 tDCS protocol and current distribution

Stimulation was delivered using a battery-powered device (Eldith DC-Stimulator-Plus, Neuroconn, Germany). Two rubber electrodes (5 x 5 cm) were placed in saline-soaked sponges and attached to the scalp using rubber bands. For prefrontal stimulation the anodal electrode was placed over the left DLPFC while the cathodal electrode was placed over the right DLPFC (F3 and F4, respectively, according to the 10/20 system of electrode placement). For bilateral stimulation of M1, the anode was centred over the hand area of left primary motor cortex, 5 cm lateral to the vertex, and the cathode over the homologous region of the right hemisphere. In the active tDCS conditions, stimulation was delivered at 2 mA for 20 minutes, with 10s ramping-up and ramping-down. In the sham tDCS conditions, participants received 30s of direct current followed by impedance control with a small current pulse being produced every 550 ms (110 μA over 15 ms), resulting in an instantaneous current of no more than 2 μA. Double-blinding was implemented through the use of a study mode on the tDCS device. SimNIBS (Thielscher, Antunes, & Saturnino, 2015) and Gmsh software (Geuzaine & Remacle, 2008) were used to visualize the spatial distribution of the electrical field induced in the brain by the DLPFC and M1 tDCS electrode montages (see Figure 2 C. and 2.D).

### 2.6 Computational modelling

In line with previous studies utilising the IBLT (Pulcu & Browning, 2017; Pulcu et al., 2019) we analysed choice behaviour with a model in which a Rescorla-Wagner learning rule (Rescorla & Wagner, 1972) was coupled to a softmax function. The model calculates the probability estimates for the win (*rwin*) and loss (*rloss*) outcomes being associated with shape ‘A’ on the next trial (*i*+1):

(1) $$\begin{array}{c} rwin_{\left(i+1\right)}=rwin_{\left(i\right)}+\alpha win*\varepsilon_{win(i)}\\ rloss_{\left(i+1\right)}=rloss_{\left(i\right)}+\alpha loss*\varepsilon_{loss\;(i)} \end{array}$$

in which *awin* and *aloss* represent the learning rates (value between 0 and 1), and *ε*_*win*(*i*)_ and *ε*_*loss*(*i*)_ represent the prediction error for the win and loss outcomes on the *i*^th^ trial, respectively. The probability estimates for wins and losses are updated on every trial for both shapes, independent of whether the shape was chosen or not. *rwin* and *rloss* were initialised at 0.5 at the start of each block. Estimated outcome probabilities were then transformed into a single choice probability:

(2) $$P_{\left(choice=A\left(i\right)\right)}=\frac{1}{1+\exp^{\left(-\beta *\left(rwin_{\left(i\right)}+t-rloss_{\left(i\right)}\right)\right)}}$$

**Figure S5:**
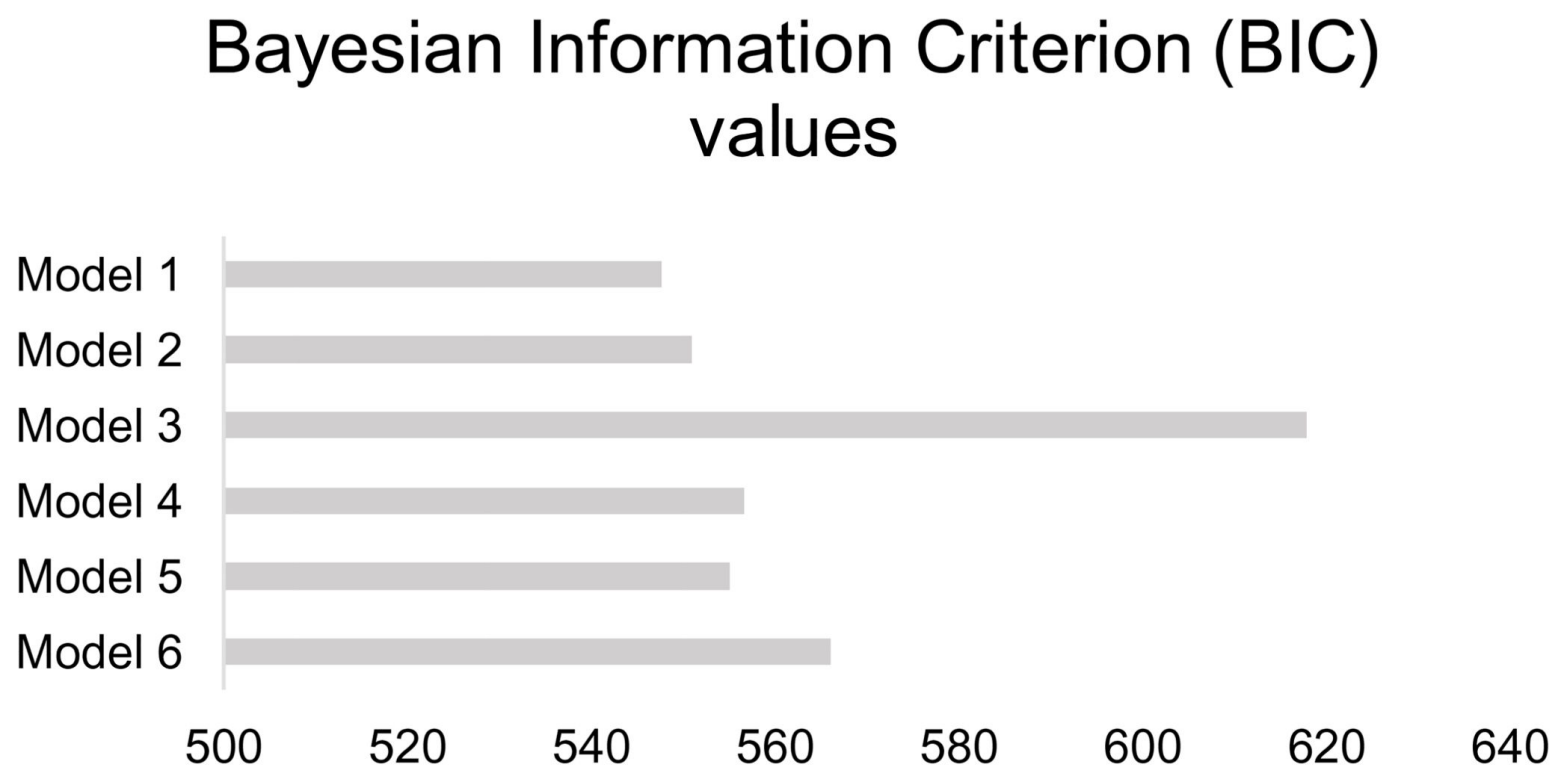
Formal comparison of computational model fit. Bars represent the sum of Bayesian Information Criterion (BIC) values per participant for each model. Smaller BIC values are indicative of a better model fit.

Here, *P*_(*choice*=*A*(*i*))_ is the probability of choosing shape ‘A’ in trial *i. β* represents the inverse decision temperature, or the degree to which the expected values are used to determine choice of a particular shape. Finally, *t* reflects a potential bias towards one of the options over the other. Learning rates and *β*-values were estimated separately for each task block and participant. This was achieved by calculating the full joint posterior probability of the parameters given participants’ choices, deriving the expected value of each parameter from their marginalised probability distributions. The first 10 trials of each block were omitted when fitting the model parameters to participants’ choices, as initial learning rates without prior knowledge were expected to differ from informed learning rates in later trials of the task (Browning et al., 2015; Pulcu & Browning, 2017). The choice of this model was based on a formal comparison using Bayesian information criterion (BIC) values for five competitor models (see Extended Data Figure S5). Models were implemented using MATLAB version R2018a (The MathWorks, Inc., Natick, MA).

### 2.7 Non-computational choice behaviour

To test for behavioural effects of tDCS without reliance on the specific assumptions of the model described above, we also conducted non-computational analyses of choice behaviour on the IBLT. In these analyses we focused specifically on choices following trials where one shape was associated with both a win and a loss outcome. On such trials there is no change in earnings because the win and loss cancel each other out. Therefore, the shape selected on the next trial provides a measure of the *relative* influence of positive versus negative outcomes on participants’ subsequent choice behaviour. If the positive outcome more strongly influences a participant’s choices, they would be expected to stay with the shape currently associated with both outcomes and choose it again on the next trial. By contrast, if the negative outcome is more influential, the participant would be expected to switch on the next trial and instead choose the other shape that was not associated with both outcomes. The proportion of trials in which the participants chose the win- over the loss-driven option was calculated for each block (there were 39 or 40 of such choices per block, depending on the condition and protocol). Trials in which the win and loss outcome were associated with different shapes were excluded from the non-model-based analyses, as in these trials both outcomes promote the same choice (i.e. selecting the shape associated with the win outcome).

### 2.8 Statistical analyses

All analyses were completed in R software (Version 3.6.0). Learning rates and inverse temperature values derived from the computational model, non-computational choice behaviour, and total winnings (in £) were entered into repeated-measures ANOVAs with the ‘ezANOVA’ function from the ‘ez’ R package. The first set of analyses assessed effects of prefrontal tDCS applied during task performance on reward learning (Study 1). Next, we carried out control comparisons to confirm whether tDCS outcomes were timing- and site-specific. To contrast the effect of tDCS between studies, we conducted ANOVAs on combined datasets (Study 1 and 2, or Study 1 and 3) and included Cognitive State (‘tDCS during task’ vs. ‘tDCS before task’, i.e. Study 1 vs. Study 2) or tDCS Target (DLPFC vs. M1, i.e. Study 1 vs. Study 3) as a between-subjects variable. To test for the effect of offline bifrontal tDCS and M1 tDCS, ANOVAs were also conducted separately within these datasets. Finally, data from Study 4 (replication study) were analysed in a separate ANOVA to establish whether effects of prefrontal stimulation during task performance were robust.

As in previous work (Pulcu et al., 2019) outcome measures from the Win- and Loss-volatile blocks of the IBLT were the primary focus of analysis. The main outcome of interest was participants’ learning behaviour in the Win- and Loss-volatile blocks, which were tested for outcome- and volatility-dependent effects of stimulation. The independent variables were tDCS, Outcome, Time (during/post-tDCS = blocks 2-3/4-5), and Volatility (Win-volatile/Loss-volatile blocks). The dependent variable was learning rate.

Both-volatile blocks were analysed to test for potential changes in learning from the baseline pre-tDCS block (block 1) to the final block post-tDCS/task (block 6). The independent variables were tDCS (active/sham), Outcome (win/loss), and Time (block 1/block 6). Baseline learning rates for wins and losses from Block 1 of the first of the two test sessions were included as a covariate in all analyses on learning rates to account for individual differences in baseline learning rates.

Inverse temperature values, non-model-based choice behaviour, and total winnings were also investigated with repeated-measures ANOVAs, with the independent variables of tDCS, Time, and Volatility. Stimulation order (sham or active tDCS first) was included as a between-subjects variable in all analyses. Effect sizes for all ANOVAs are reported as generalised eta squared values (*η*^2^_G_). Significant interactions were followed up with *post hoc* paired *t*-tests, with effect sizes reported as Cohen’s *dz*. Learning rates were transformed onto the infinite real line using an inverse logistic transform, and inverse temperature values were normalised with a log transformation consistent with previous studies (Pulcu & Browning, 2017; Pulcu et al., 2019). Figures and reported values represent raw parameter values to facilitate interpretation of the results. Mean and standard deviations for all model parameters are reported in the supplementary material (Extended Data Table S3).

## 3 Results

### 3.1 Prefrontal tDCS during task performance increased reward learning rates

#### 3.1.1 Computational parameters

**Figure S6:**
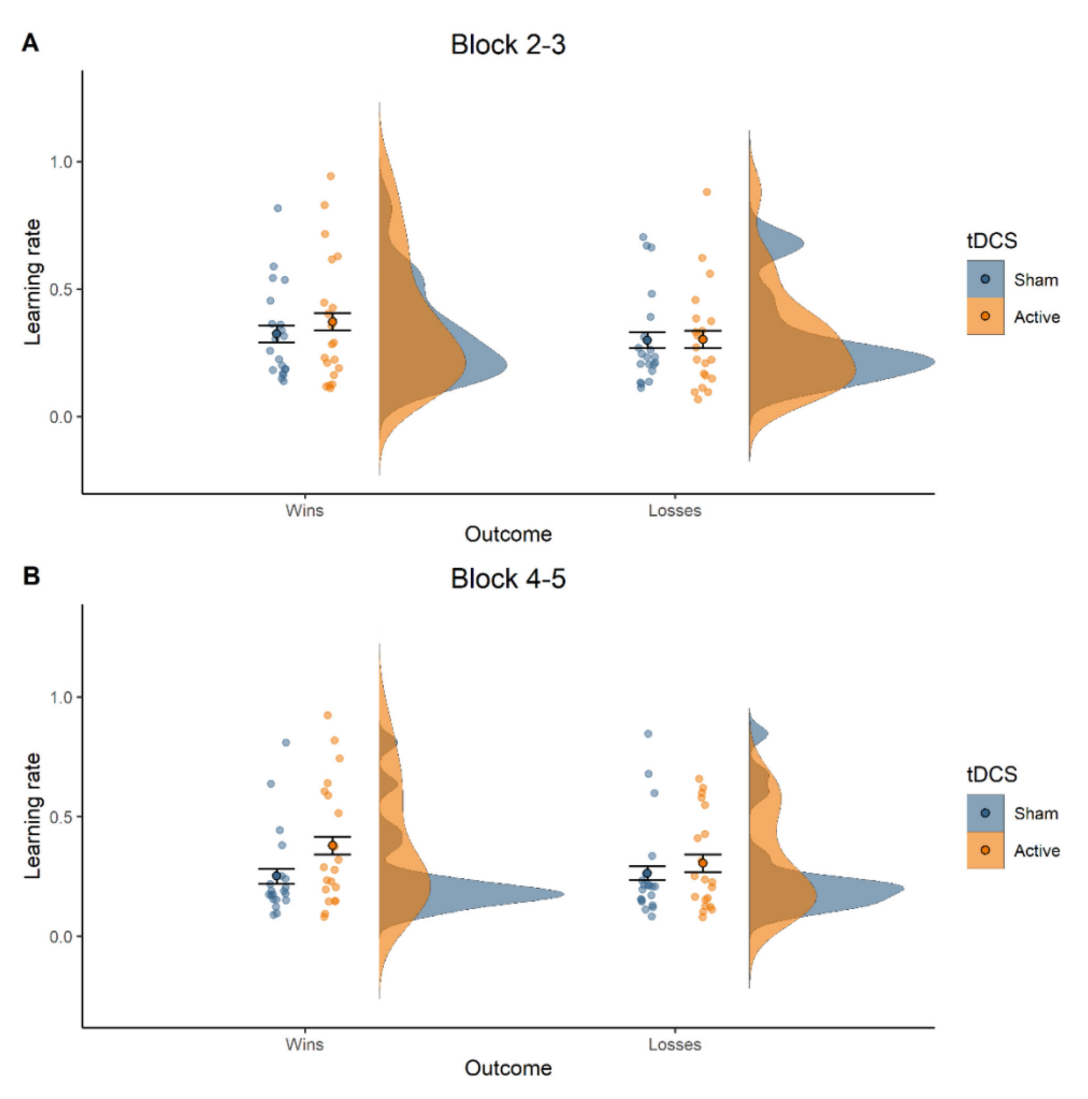
Stable increase in reward learning rates with online prefrontal tDCS. (**A**) Learning rates in Blocks 2-3 (during tDCS). (**B**) Learning rates in Blocks 4-5 (after tDCS). Violin-plots show the distribution of learning rates by tDCS condition (sham = blue, active = orange). Summary statistics are provided in boxplots, with the black horizontal line indicating the median and whiskers representing the 25^th^ and 75^th^ percentiles of values. Dots represent participants’ individual data points averaged across task blocks.

We predicted that bifrontal tDCS applied during task performance would lead to a relative increase in reward versus punishment learning rates, i.e. increase reward or decrease punishment learning rates. In line with this hypothesis, we observed a valence-specific effect of prefrontal tDCS across the Win- and Loss-volatile blocks (tDCS x Outcome interaction: *F*_(1,18)_ = 4.89, *p* = 0.040; *η*^2^_G_ = 0.006). Active tDCS caused higher learning rates for win (*t*_(19)_ = 2.11, *p* = 0.048, *dz* = 0.472) but not loss outcomes (*t*_(19)_ = 0.35, *p* = 0.728). This effect did not change over time (tDCS x Outcome x Time interaction: *F*_(1,18)_ = 0.56, *p* = 0.464), indicating that the increase in reward learning rates was maintained for at least 15 minutes after tDCS offset (see Extended Data Figure S6. for visualization of learning rates over time).

**Figure 3:**
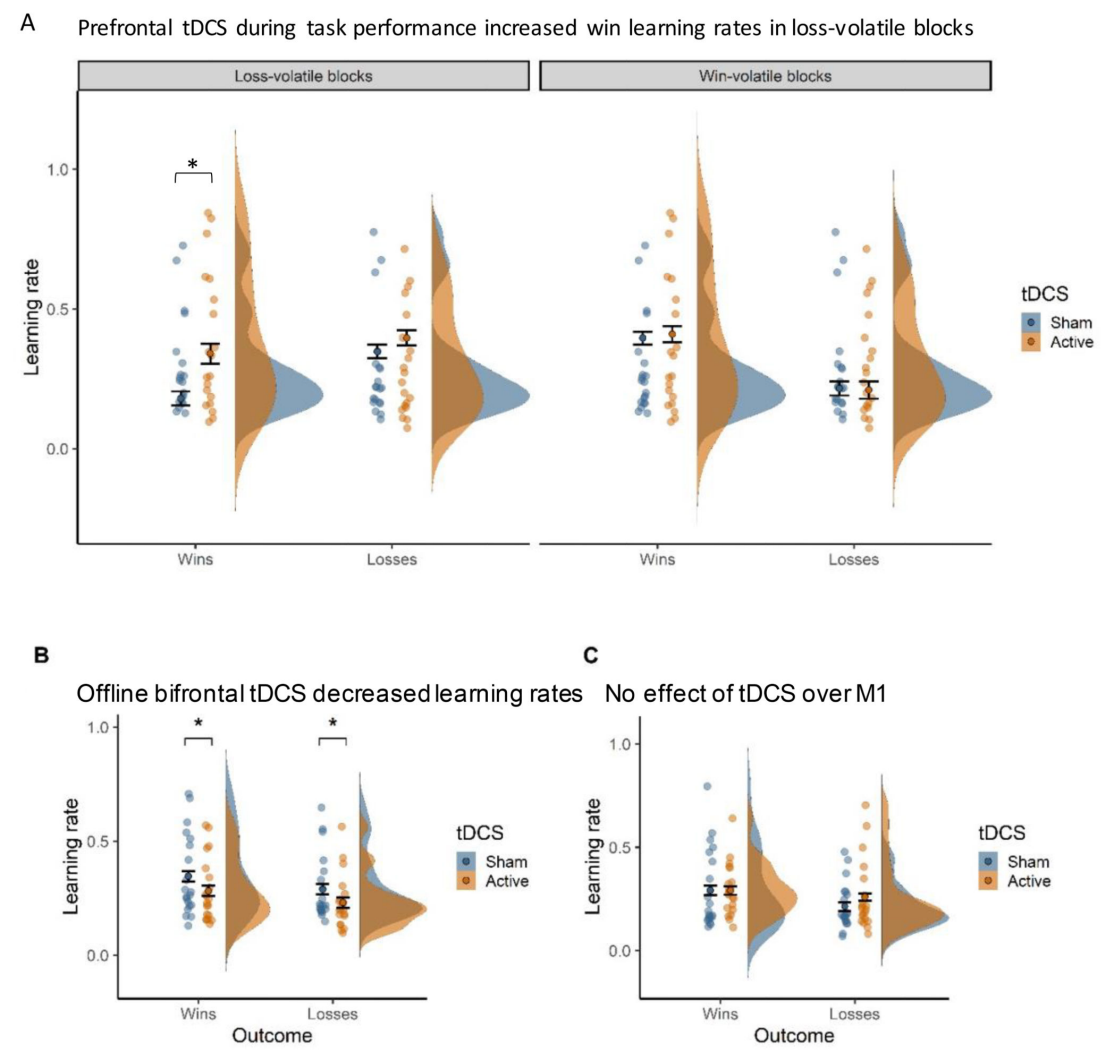
Prefrontal tDCS during task performance selectively increased win learning rates. **(A)** Prefrontal stimulation *during* task performance selectively increased learning rates for win outcomes in blocks where losses were volatile (*p <0.05). **(B)** The valence-specific effect shown in A) was also cognitive-state specific, as prefrontal stimulation applied *before* task performance decreased both win and loss learning rates (*p <0.05). **(C)** The valence-specific effect shown in A) was also anatomically specific, as stimulation over motor cortex (M1) had no such effect. Violin-plots show the distribution of learning rates by tDCS condition (sham = blue, active = orange). Dots and error bars represent the mean and SEM values. Jittered dots show participants’ individual data points averaged across task blocks.

Stimulation effects also varied by volatility (tDCS x Volatility interaction: *F*_(1,18)_ = 8.09, *p* = 0.011, *η*^2^_G_ = 0.014), such that tDCS increased learning rates in Loss-volatile (*t*_(19)_ = 2.47, *p* = 0.023, *dz* = 0.551) but not Win-volatile blocks (*t*_(19)_ = -0.12, *p* = 0.905). Although the tDCS Condition x Outcome x Volatility interaction effect was not significant (*F*(1,18) = 2.40, *p* =.138, *η*^2^_G_ = .002), the two observed interaction effects suggest that bifrontal compared to sham tDCS only increased win learning rates in blocks where losses were volatile (see figure 3A for a visualisation of the 3-way interaction). To verify this, we ran an ANOVA including only the win learning rate. This showed that the effect of tDCS on the win learning rate depended on Volatility (tDCS Condition x Volatility: *F*(1,18), *p* = .008, *η*^2^_G_ = .031). Paired t-tests confirmed that tDCS increased win learning rates in the losses-volatile (*t*(19) = 2.88, *p* = .009, *dz* = .517) but not in the wins-volatile block (*t*(19) = 0.24, *p* = .806).

Prefrontal stimulation specifically altered learning rates, without changing the randomness of participants’ choices (no effect of tDCS on inverse temperature parameters; all *p* >0.05; see Extended Data for analysis of inverse temperature and bias term). For the Both-volatile blocks (Block 1 vs 6), there was no difference in learning rates or inverse temperature values between the active versus sham tDCS sessions (all *p* > 0.05).

#### 3.1.2 Non-computational choice behaviour and total winnings

In addition to the computational analyses, we assessed simple choice behaviour to investigate tDCS effects in the absence of assumptions associated with the computational models. Here, we focused on participants’ choices following trials where one shape was associated with both the win and the loss outcome. We calculated the proportion of ‘win-driven’ choices (i.e., the participant selected the shape which was associated with both outcomes in the previous trial). For these win-driven choices, there was an interaction of tDCS and volatility (*F*_(1,18)_ = 7.90, *p* = 0.016, *η*^2^_G_ = 0.029). Active stimulation increased the number of win-driven choices in Loss-volatile blocks (*t*_(19)_ = 3.33, *p* = 0.004, *dz* = 0.745), with no effect in Win-volatile blocks (*t*_(19)_ = -0.71, *p* = 0.489). Thus, in line with the computational modelling results, tDCS to DLPFC increased win learning rates when win outcomes were less informative than loss outcomes (i.e. in the Loss-volatile blocks). For total winnings, we found a main effect of tDCS (*F*_(1,18)_ = 5.91, *p* = 0.026, *η*^2^_G_ = 0.023). Overall, participants won less money with active than sham stimulation. This decrease in total winnings is consistent with participants’ increasing reliance on uninformative rewards. In the Both-volatile blocks, there were no effects of tDCS (all *p* >0.05).

### 3.2 Opposing effects of tDCS applied during vs. before task performance

To test for cognitive state specificity, in Study 2 we applied prefrontal tDCS prior to task performance while participants simply rested. To test whether the effect of online bifrontal tDCS on learning rates significantly differed from the effect of offline bifrontal tDCS we conducted an ANOVA on the combined dataset (i.e. Study 1 and Study 2, with Cognitive State as the between-subject factor). Crucially, tDCS applied during or before task performance had diverging effects on win and loss learning rates (Cognitive State x tDCS x Outcome interaction: *F*_(1,36)_ = 5.10, *p* = 0.030, *η*^2^_G_ = 0.003). In contrast with the specific increase in reward learning with tDCS *during* task performance, tDCS *before* task performance induced a *reduction* of both win and loss learning rates (ANOVA within offline bifrontal tDCS dataset (Study 2), *t*_(19)_ = -2.18, *p* = 0.042, *dz* = 0.487) (Figure 3B). Contrary to tDCS *during* task performance, tDCS *before* task performance did not affect any of the non-computational outcomes (ANOVA within offline bifrontal tDCS dataset (Study 2), all *p* >0.05). As predicted, the specific effects of prefrontal tDCS on reward learning were induced only by tDCS *during* task performance.

### 3.3 Anatomical specificity of tDCS effects

To determine the anatomical specificity of tDCS effects, we compared learning rates with DLPFC (during task) versus M1 stimulation (ANOVA on combined datasets from Study 1 and Study 3 with tDCS Target as between-subject factor). Importantly, stimulation effects differed by outcome for the two tDCS targets in Win- and Loss-volatile blocks (tDCS Target x tDCS x Outcome interaction: *F*_(1,36)_ = 4.29, *p* = 0.045, *η*^2^_G_ = 0.005) (Figure 3C). Whereas prefrontal stimulation specifically increased win learning rates, tDCS over motor cortex had no effect on learning rates for either wins (*t*_(19)_ = 0.22, *p* = 0.828) or losses (*t*_(19)_ = 1.47, *p* = 0.159)(ANOVA within the M1 stimulation dataset (Study 3)). Thus, the valence-specific increase in reward learning observed with prefrontal stimulation was anatomically specific. In addition, tDCS effects varied depending on the volatility of the outcomes (tDCS Target x tDCS x Volatility interaction: *F*_(1,36)_ = 13.58, *p* <0.001, *η*^2^_G_ = 0.012). Whereas prefrontal tDCS increased learning rates in the Loss-volatile blocks, M1 stimulation increased learning rates in the Win-volatile blocks (ANOVA within the M1 stimulation dataset (Study 3), *t*_(19)_ = 2.40, *p* = 0.027, *dz* = 0.537). As expected, tDCS over M1 did not alter any of the non-computational outcomes (ANOVA within the M1 stimulation dataset (Study 3), all *p* >0.05).

### 3.4 Replication of the effects of tDCS applied during task performance

**Figure 4:**
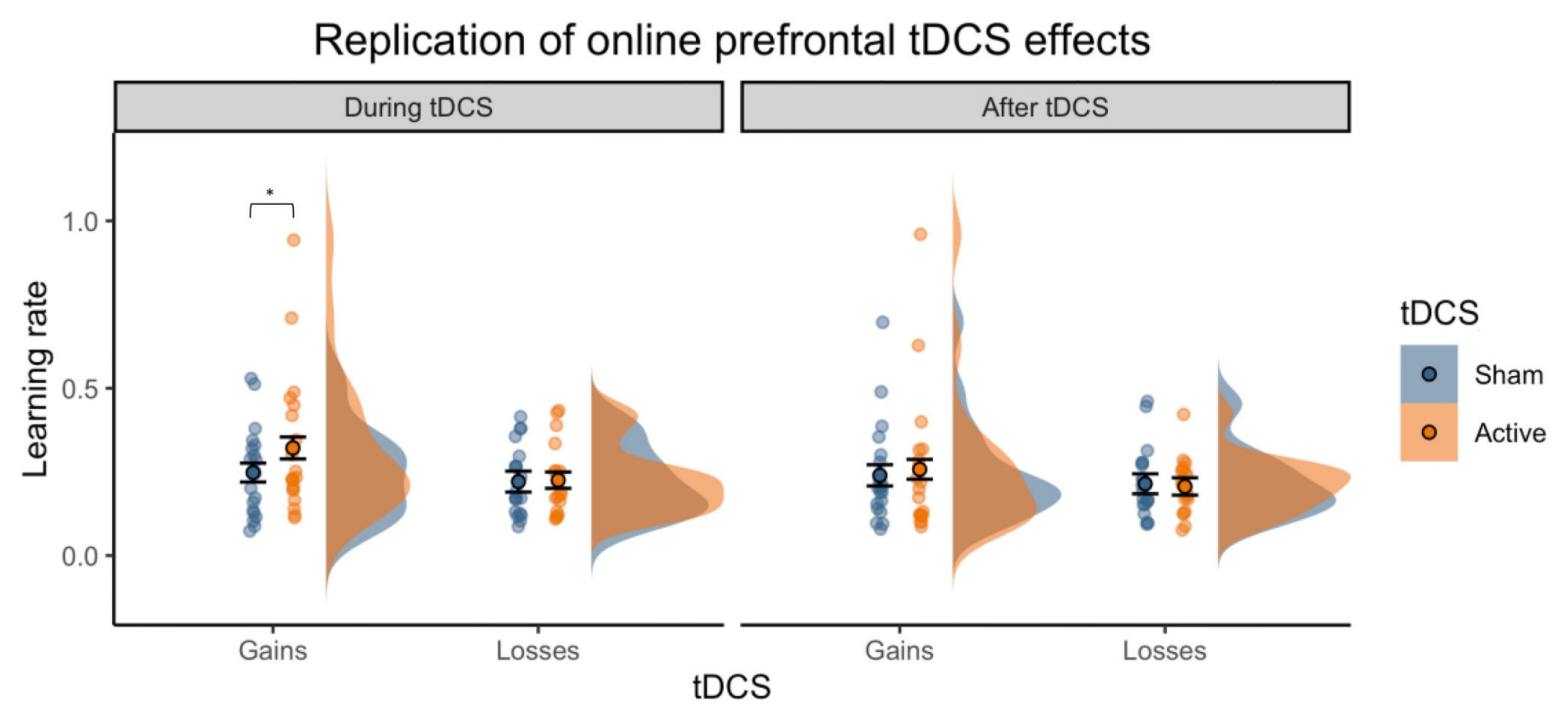
Replication study. Planned comparisons in the replication study (Study 4) revealed that prefrontal tDCS during task performance induced a marginally significant increase in win learning rates, but only during the stimulation period (. *=* 0.05), replicating the key finding of Study 1 (Figure 3). Violin-plots show the distribution of learning rates by outcome (wins = blue, losses = orange). Dots and error bars represent the mean and SEM values. Jittered dots show participants’ individual data points averaged across task blocks.

In Study 4, we carried out a replication of Study 1 to determine whether prefrontal tDCS during task performance consistently increases reward learning rates. This dataset was analysed in a separate ANOVA. We did not find the expected outcome valence- or volatility-specific effects of tDCS observed in Study 1 (tDCS x Outcome interaction: *F*_(1,18)_ = 1.30, *p* = 0.269; tDCS x Volatility interaction: *F*_(1,18)_ = 0.01, *p* = 0.930). There was also no significant interaction of tDCS condition with volatility in the non-computational analyses (*F*_(1,18)_ = 0433, *p* = 0.519). However, to specifically test whether the increase in win learning rates in study 1 replicated, we also conducted planned comparisons between real and sham tDCS. *During* stimulation, real compared to sham tDCS induced a marginally significant increase in win learning rates (*t*(19) = 2.08, *p* = .050, *dz* = 0.387). Unlike in study 1, this effect did not persist after the stimulation (*t*(19) = 0.17, *p* = .86)(Figure 4). As in study 1, there was no significant effect on loss learning rates neither during tDCS nor after (during tDCS: *t*(19) = 1.1, *p* = .28; after tDCS: *t*(19) = - 0.62, *p* = .52). Altogether, this suggests that active prefrontal stimulation was associated with increased learning rates for positive outcomes compared to sham tDCS, although the effect was weaker in the replication dataset than in the original Study 1.

## 4 Discussion

This study tested the malleability of reward learning with transcranial direct current stimulation in healthy adults. Prefrontal tDCS applied during task performance selectively increased reward learning rates. A replication study demonstrated a weaker but similar effect of prefrontal tDCS on reward learning rates. As predicted, tDCS effects were cognitive-state dependent, occurring only when tDCS was applied *during* task performance. When tDCS was applied before task performance learning rates for both wins and losses *decreased*. In addition, the effects of tDCS applied during task performance were anatomically specific, with no effects of motor cortex stimulation on the balance of reward and loss learning rates. Taken together, these findings demonstrate the potential of prefrontal tDCS during task performance to change the relative influence of positive and negative outcomes on choice behaviour.

### 4.1 Valence-specific tDCS effects

In study 1 we found that bifrontal tDCS applied during reinforcement learning increased reward learning rates. There was evidence that the effect was only present when losses were volatile, i.e. when wins were uninformative. In our replication study, the interaction between tDCS and Outcome (win vs. loss learning rate) was not significant but direct comparisons between real and sham tDCS indicated that tDCS marginally increased win but not loss learning rates during the stimulation period. Although the effect in the replication sample was weaker, this finding increases our confidence that the increase in win learning rate might be a reliable effect. In the replication sample, there was no evidence that the tDCS effect depended on the Volatility condition. We therefore limit our discussion to the increase in win learning rate (independent of Volatility).

Our finding of increased learning that was specific for positive outcomes is consistent with several prior tDCS studies targeting left DLPFC, which have reported improvements in cognitive control for positive stimuli (Vanderhasselt et al., 2013) and improved recognition of positive emotions (M. A. Nitsche et al., 2012). According to the asymmetry hypothesis of valence, responses to positive and negative stimuli are lateralised to left and right DLPFC, respectively (Davidson, 1992; Herrington et al., 2010; Herrington et al., 2005; Wyczesany, Capotosto, Zappasodi, & Prete, 2018). Anodal (excitatory) stimulation of left DLPFC (and here also concurrent cathodal (inhibitory) stimulation of right DLPFC) may therefore shift the relative balance of information processing in favour of positive versus negative stimulus information processing. Since the reversal of negative biases is thought to be one key mechanism underlying effective antidepressant treatments (Godlewska et al., 2016; Godlewska, Norbury, Selvaraj, Cowen, & Harmer, 2012; Harmer, Goodwin, & Cowen, 2009), the effects observed in this study might be of potential interest in depression treatment.

Negative biases in depression are hypothesised to arise from overestimation of information content of negative relative to positive events, and a consequent relative increase in punishment compared to reward learning rates (Pulcu & Browning, 2017, 2019). In our study, bifrontal tDCS selectively increased reward learning rates, which suggests that individuals estimated positive outcomes to be more informative. This effect might be beneficial in depression treatment to counter-balance negative cognitive biases. For example, if an individual estimates positive events to be equally informative as negative events, they will consider positive and negative feedback to the same extent and will give up pursuing a goal less quickly (i.e. equal learning rates for positive and negative outcomes).

Bifrontal tDCS led to less winnings in our study. In healthy individuals, the increase in reward learning might have been suboptimal since the optimal learning rate depends on the information content of outcomes (i.e. higher is not always better). However, in populations with aberrant learning rates (e.g. negative bias in anxiety/depression), the increase in reward learning rates could be beneficial in two ways. First, it might help optimise choice behaviour in individuals showing decreased reward learning rates as might be the case in depression (Brown et al., 2021). Second, an increase in reward learning might counter-act negative biases by re-balancing positive versus negative information processing (see above). We intend to investigate this in future work.

### 4.2 Neural mechanisms

Our study was designed to test whether bifrontal tDCS, which is commonly applied in depression trials, can modulate learning rates in a reinforcement learning task. Given that this setup stimulates wide regions in the frontal cortex, it is difficult to draw conclusions about the exact anatomical substrate. Increasing evidence suggests that the DLPFC is involved in adjusting learning rates to volatility (Farashahi et al., 2019; Massi et al., 2018). The observed effect might also be caused by excitability changes in the dorsal anterior cingulate, an anatomically connected area which has been shown to be activated in response to volatility (Behrens et al., 2007). Electric field modelling suggests that the highest field strength might actually be generated in between rather than underneath the electrodes (Karabanov, Saturnino, Thielscher, & Siebner, 2019), i.e. in the medial prefrontal cortex. Previous studies have linked the medial prefrontal cortex (mPFC) with various aspects of reward learning, including action-outcome predictions (Alexander & Brown, 2011), social prediction errors (Behrens, Hunt, Woolrich, & Rushworth, 2008), and belief updating (Kuzmanovic, Rigoux, & Tittgemeyer, 2018). Concurrent tDCS-fMRI studies are required to establish the nature of functional brain changes underpinning the behavioural effects observed here. Such an approach could reveal the key brain regions mediating the tDCS-induced increase in win learning rate and enable follow-on more targeted tDCS montages, e.g. high-definition tDCS, in the future.

### 4.3 Cognitive-state dependency of tDCS effects

The valence-specific effect of bifrontal tDCS applied during task performance was cognitive-state dependent. In contrast to bifrontal tDCS during task performance, bifrontal tDCS *before* task performance led to decreased learning rates in general. This is consistent with the known brain-state dependency of non-invasive brain stimulation effects (Li et al., 2019; Silvanto, Muggleton, & Walsh, 2008). Although we do not fully understand why tDCS before task performance caused a decrease in learning rates, differential effects of tDCS during versus before task performance have been observed previously (Li et al., 2019; Stagg et al., 2011) and are hypothesised to depend on metaplastic mechanisms (Müller-Dahlhaus & Ziemann, 2015).

### 4.4 Limitations and future directions

**Figure S7:**
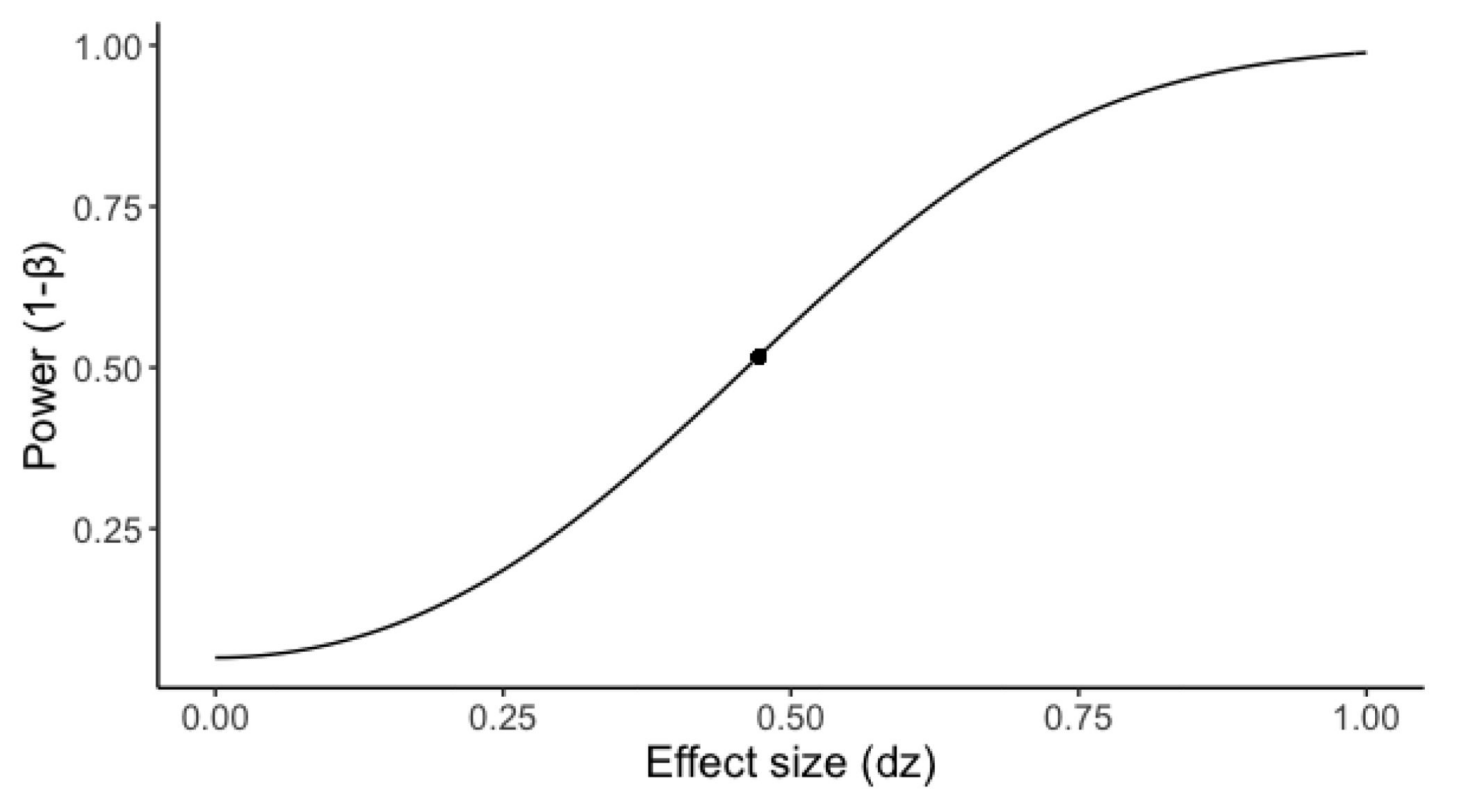
Sensitivity power analysis for a paired t-test with a sample size of N = 20. The dot represents the effect size observed in Study 1 (*dz* = 0.472) and the resulting power (1-β = 0.51).

This proof-of-concept study provides promising evidence for the potential utility of prefrontal tDCS in altering reward learning rates relevant to affective disorders (Browning et al., 2015; Gagne et al., 2020; Pulcu & Browning, 2019). However, this work has limitations which should be addressed in future studies. The sample size of 20 participants per study (real vs. sham tDCS in a within-subjects design) was chosen pragmatically based on financial and time resource constraints. During the study design phase there were no prior reports of valence-specific effects of tDCS on learning rates that could have informed effect size estimates for a priori power analysis. Post hoc sensitivity analysis of the effect size observed in Study 1 (*dz* = 0.472) indicated that the power level achieved with 20 participants was low (0.51)(see Extended Data Figure S7 for sensitivity power analysis). Low power increases the risk of false-positive findings (Christley, 2010). To address this limitation, we conducted Study 4 and recruited an independent sample of another 20 participants to test whether our findings would replicate. Study 4 yielded a partial replication of Study 1. The key finding of Study 1, that stimulation selectively increased win learning rates, was also observed in the replication study. However, in Study 4 the effect was limited in duration (i.e. it did not outlast tDCS offset), and there was no change in win-driven choice behaviour. This suggests a relatively weaker effect of tDCS in the replication study, as non-model-based outcomes are less powerful for detecting behavioural changes than the computational parameters. Although the power level achieved in these two studies was rather low, the joint probability of observing the same tDCS effect (specific increase in win learning rate) across both studies is very low, making it unlikely that this finding is a false-positive. Additional independent replications would be useful to further confirm the tDCS effect reported here.

A logical next step within this line of research will be to determine whether the present findings can be extended to clinical groups. All tDCS effects observed here were in healthy adults with low scores on measures of depression or anxiety. Compared to healthy adults, individuals with depression tend to be less sensitive to rewards (Eshel & Roiser, 2010) and present with hypoactivity of left DLPFC (Disner et al., 2011; Grimm et al., 2008). Such baseline differences in neural and cognitive patterns are likely to lead to different effects of prefrontal tDCS from those observed here on learning performance. Therefore, we are planning to conduct a study with individuals who have depressive symptoms using the same experimental paradigm. We will first test whether their task performance differs from healthy individuals (e.g. increased punishment vs. reward learning rates) and then assess if/how stimulation interacts with their behaviour on this task.

Finally, we did not examine transfer of tDCS effects. Future studies will need to address the potential generalisation of changes in reinforcement learning to different tasks and/or to clinically relevant outcomes (e.g. (Overman, Browning, & O’Shea, 2020)).

### 4.5 Conclusion

Using a computational approach, we demonstrated that reward learning rates can be selectively increased by tDCS applied to bilateral DLPFC during learning. tDCS effects were cognitive state-dependent, such that learning rates were increased with tDCS applied during, but decreased with tDCS applied before task performance. These results provide preliminary evidence that prefrontal tDCS applied during task performance can alter cognitive mechanisms thought to be relevant to maintaining symptoms of affective disorders. In the future, we aim to investigate whether this tDCS paradigm can be used to re-shape reward learning processes and thus potentially ameliorate clinical symptoms in individuals with anxiety and depression.

## Extended Data

**Table S2:** Mean (SD) PANAS and STAI-state scores by tDCS condition

	Study 1 Online DLPFC (N = 20)		Study 2 *Offline DLPFC* *(N = 20)*		Study 3 *Online M1* *(N = 20)*		Study 4 *Replication* *(N = 20)*	
***PANAS Positive***								
Sham tDCS	26.8 (4.9)	22.4 (6.2)	28.2 (6.4)	26.0 (6.4)	32.5 (7.3)	30.4 (8.7)	31.0 (7.10)	27.4 (7.3)
Active tDCS	27.2 (5.3)	24.4 (6.6)	28.6 (6.3)	24.4 (6.0)	31.3 (7.9)	28.1 (8.6)	30.6 (7.0)	27.7 (8.6)
***PANAS Negative***								
Sham tDCS	10.9 (1.1)	10.4 (0.6)	13.0 (3.0)	11.6 (2.6)	11.0 (1.9)	11.0 (1.4)	11.5 (2.0)	11.4 (2.4)
Active tDCS	11.6 (2.3)	10.6 (0.9)	12.0 (2.7)	11.9 (3.0)	11.9 (4.6)	10.8 (1.4)	11.8 (2.1)	11.1 (1.5)
***STAI-State***								
Sham tDCS	30.0 (4.7)	31.8 (6.9)	29.1 (6.7)	30.0 (7.7)	29.0 (7.3)	29.8 (6.5)	32.2 (8.7)	30.6 (8.1)
Active tDCS	30.0 (6.2)	31.9 (4.8)	30.5 (7.9)	29.3 (6.7)	30.6 (7.9)	31.8 (6.9)	29.6 (7.4)	30.8 (8.6)

*BDI-II; Beck’s Depression Inventory II, PANAS; Positive and Negative Affect Scale; STAI, State-Trait Anxiety Inventory.*

**Table S3:** Mean and standard deviation for all model parameters for studies 1-4.

Sample	tDCS Condition	Volatility	Time	Win LR mean	Win LR SD	Loss LR mean	Loss LR SD	Beta mean	Beta SD	Bias term *t* mean	Bias term *t* SD
Bifrontal online	Real	losses-volatile	First half	-0.92	2.01	-0.48	1.07	0.75	0.72	0.02	0.17
Bifrontal online	Real	losses-volatile	Second half	-1.10	1.97	-0.42	1.27	0.79	0.62	0.06	0.16
Bifrontal online	Real	wins-volatile	First half	-0.51	1.10	-1.84	1.53	0.74	0.60	-0.05	0.14
Bifrontal online	Real	wins-volatile	Second half	-0.21	1.36	-1.86	1.54	0.81	0.48	0.01	0.17
Bifrontal online	Real	both-volatile	First half	0.14	1.17	-0.82	1.36	0.84	0.45	0.00	0.13
Bifrontal online	Real	both-volatile	Second half	-0.49	1.40	-1.37	1.40	0.90	0.53	-0.05	0.12
Bifrontal online	Sham	losses-volatile	First half	-1.69	1.18	-0.64	0.97	0.88	0.64	0.02	0.09
Bifrontal online	Sham	losses-volatile	Second half	-2.07	1.15	-0.76	0.94	0.93	0.56	0.05	0.10
Bifrontal online	Sham	wins-volatile	First half	-0.17	1.15	-1.57	1.46	0.92	0.48	0.03	0.14
Bifrontal online	Sham	wins-volatile	Second half	-0.66	1.02	-1.92	1.50	0.94	0.54	-0.09	0.13
Bifrontal online	Sham	both-volatile	First half	-0.36	1.38	-0.91	1.36	0.93	0.39	-0.03	0.15
Bifrontal online	Sham	both-volatile	Second half	-0.95	1.38	-1.06	1.21	1.02	0.47	0.06	0.14
Bifrontal offline	Real	losses-volatile	First half	-1.69	1.44	-1.07	0.87	0.99	0.64	0.01	0.12
Bifrontal offline	Real	losses-volatile	Second half	-1.62	1.45	-0.98	1.25	1.05	0.64	0.00	0.12
Bifrontal offline	Real	wins-volatile	First half	-0.64	0.86	-1.72	1.28	1.18	0.38	0.00	0.11
Bifrontal offline	Real	wins-volatile	Second half	-0.79	0.85	-2.17	1.04	1.01	0.46	-0.01	0.09
Bifrontal offline	Real	both-volatile	First half	-0.61	1.01	-0.65	1.15	1.11	0.32	-0.01	0.16
Bifrontal offline	Real	both-volatile	Second half	-0.92	0.98	-1.55	1.66	1.11	0.41	0.04	0.13
Bifrontal offline	Sham	losses-volatile	First half	-1.21	1.27	-0.67	0.64	0.81	0.72	0.03	0.21
Bifrontal offline	Sham	losses-volatile	Second half	-1.07	1.35	-0.67	0.84	0.88	0.56	0.00	0.27
Bifrontal offline	Sham	wins-volatile	First half	-0.34	1.00	-1.97	1.43	0.94	0.55	-0.03	0.19
Bifrontal offline	Sham	wins-volatile	Second half	-0.50	1.06	-1.42	1.39	0.77	0.70	-0.03	0.18
Bifrontal offline	Sham	both-volatile	First half	-0.33	1.56	-0.21	1.30	0.87	0.59	0.04	0.12
Bifrontal offline	Sham	both-volatile	Second half	-0.79	1.40	-0.87	1.63	0.94	0.65	0.04	0.15
Motor cortex online	Real	losses-volatile	First half	-1.61	1.04	-0.83	0.70	0.95	0.39	0.07	0.14
Motor cortex online	Real	losses-volatile	Second half	-1.62	1.21	-0.95	1.05	1.02	0.41	0.02	0.14
Motor cortex online	Real	wins-volatile	First half	-0.55	0.77	-1.60	1.48	1.03	0.49	0.01	0.11
Motor cortex online	Real	wins-volatile	Second half	-0.57	0.95	-1.93	1.41	1.05	0.60	-0.02	0.12
Motor cortex online	Real	both-volatile	First half	0.09	1.19	-1.28	1.31	0.98	0.43	-0.03	0.08
Motor cortex online	Real	both-volatile	Second half	-0.47	1.26	-1.29	1.85	1.08	0.46	-0.03	0.17
Motor cortex online	Sham	losses-volatile	First half	-1.11	1.91	-0.80	0.88	0.76	0.63	0.10	0.15
Motor cortex online	Sham	losses-volatile	Second half	-1.65	1.42	-1.16	0.70	0.91	0.73	-0.02	0.17
Motor cortex online	Sham	wins-volatile	First half	-0.73	1.05	-2.03	1.17	1.05	0.58	-0.03	0.11
Motor cortex online	Sham	wins-volatile	Second half	-1.03	1.06	-2.54	1.03	1.08	0.43	0.00	0.12
Motor cortex online	Sham	both-volatile	First half	-0.51	1.75	-0.96	1.59	1.05	0.42	-0.01	0.08
Motor cortex online	Sham	both-volatile	Second half	-0.32	1.09	-1.09	1.23	1.01	0.48	0.00	0.17
Replication bifrontal online	Real	losses-volatile	First half	-1.42	1.62	-0.95	0.86	0.97	0.48	0.05	0.16
Replication bifrontal online	Real	losses-volatile	Second half	-1.87	1.57	-0.84	0.51	1.02	0.45	0.04	0.12
Replication bifrontal online	Real	wins-volatile	First half	-0.46	1.14	-1.97	0.87	0.96	0.53	-0.07	0.10
Replication bifrontal online	Real	wins-volatile	Second half	-0.79	1.27	-2.56	1.03	1.17	0.35	-0.02	0.09
Replication bifrontal online	Real	both-volatile	First half	-0.45	1.44	-2.14	1.50	1.17	0.36	-0.03	0.10
Replication bifrontal online	Real	both-volatile	Second half	-1.26	1.23	-1.79	1.08	1.21	0.39	-0.02	0.10
Replication bifrontal online	Sham	losses-volatile	First half	-1.96	0.91	-0.90	0.93	0.91	0.53	0.04	0.10
Replication bifrontal online	Sham	losses-volatile	Second half	-1.98	1.13	-0.85	1.02	0.99	0.59	0.07	0.14
Replication bifrontal online	Sham	wins-volatile	First half	-0.77	1.22	-2.38	1.19	1.04	0.45	0.01	0.18
Replication bifrontal online	Sham	wins-volatile	Second half	-0.80	1.09	-2.36	0.88	1.08	0.35	-0.02	0.08
Replication bifrontal online	Sham	both-volatile	First half	-0.49	1.64	-0.78	1.27	1.02	0.36	-0.01	0.13
Replication bifrontal online	Sham	both-volatile	Second half	-1.04	1.34	-1.80	1.14	1.08	0.51	0.03	0.09

Note that an inverse logistic transform has been applied to the learning rate estimates (LR), and a log transform to the inverse temperature estimates (Beta).

## Supplementary Material

Extended Data

## Figures and Tables

**Table 1:** Mean (SD) baseline characteristics across studies

	Study 1 *Prefrontal tDCS* *during task* *(n = 20)*	Study 2 *Prefrontal tDCS* *before task* *(n = 20)*	Study 3 *M1 tDCS during task* *(n = 20)*	Study 4 *Replication of* *Study 1* *(n = 20)*
	*Prefrontal tDCS during task (n = 20)*	*Prefrontal tDCS before task (n = 20)*	*M1 tDCS during task (n = 20)*	*Replication of Study 1 (n = 20)*
*Sociodemographic data*				
Female (%)	9 (45.0)	15 (75.0)	14 (70.0)	7 (35.0)
Age, years	25.0 (4.3)	25.2 (5.8)	24.6 (6.4)	24.2 (3.6)
*Clinical measures*				
STAI-Trait	37.5 (8.9)	38.4 (7.9)	32.6 (8.7)	35.6 (5.9)
BDI	5.1 (5.2)	5.6 (8.0)	3.1 (3.4)	4.0 (3.8)

BDI = Beck Depression Inventory-II, score range = 0-63. STAI-Trait = State-Trait Anxiety Inventory, trait form, score range = 20-80.

## Data Availability

Raw data, analysis scripts, and task materials can be accessed on Open Science Framework: https://osf.io/av9pf/, DOI: 10.17605/OSF.IO/AV9PF.

## Highlights

- prefrontal cortex tDCS during task performance increased learning from win outcomes
- the effect was anatomically specific: motor cortex tDCS had no such effect
- the effect was cognitive state-dependent: it did not occur with tDCS before the task
- prefrontal cortex tDCS before the task decreased learning from wins and losses
- modulation of learning rates through tDCS might be beneficial in psychiatry
