# Supplementary material for: Stimulating human prefrontal cortex increases reward learning
Source: Neuroimage. 2023 May 1;271:120029. doi: 10.1016/j.neuroimage.2023.120029 (PMC11968408; doi:10.1016/j.neuroimage.2023.120029)
Supplement: Supplementary file 1 [file mmc1.docx]

Stimulating human prefrontal cortex increases reward learning

Extended Data

Model selection

BIC values were calculated for the model used for analyses (**Model 1**) and the following five alternative models:

Model 2: Rather than modelling a single inverse temperature value, it is possible that participants display different stochastic choice behaviour for the win and loss outcomes. This model, which has been applied in previous studies of the IBLT (Pulcu & Browning, 2017), therefore incorporates separate inverse temperature parameters for wins and losses as follows:

$$P_{(choice=A(i))} = \frac{1}{1+\exp^{(-\beta win * rwin_{(i)} - \beta loss * rloss_{(i)})¿}} \qquad (1)$$

Model 3: Instead of learning the independent probabilities of win and loss outcomes, participants might take a model-free approach to the task by learning an overall value of each of the presented shapes (Pulcu & Browning, 2017):

$$v^{A}{}_{(i+1)} = v^{A} + a * ¿) \qquad (2)$$

in which v^A represents the value of shape "A", α is a single learning rate for updating the value, and $out_{(i)}$ is the outcome of trial *i* (i.e., win – loss for shape "A", which can be -1, 0, or 1). In the first trial, the value of shape "A" is set at 0. The estimated values of the two

presented shapes were transformed into a choice probability by applying a softmax function using a single inverse temperature parameter.

Model 4: Similar to Model 1, this model calculates two learning rates and one inverse temperature parameter. This model is slightly simpler, however, in that it omits the "tendency" parameter *t* as described in Equation 4.

Model 5: A model using a single learning rate for win and loss outcomes combined, with two inverse temperature values for the two individual outcomes.

Model 6: The final model was similar to Model 2, with the main difference being that the values for the win and loss outcomes were centred at zero prior to multiplication with the inverse temperature values:

$$P_{(choice=A|i))} = \frac{1}{1+\exp^{¿¿}¿} \qquad (3)$$

As shown in Fig.S.1, Model 1 provided the best fit to the data across the four studies and was therefore selected for the computational analyses.

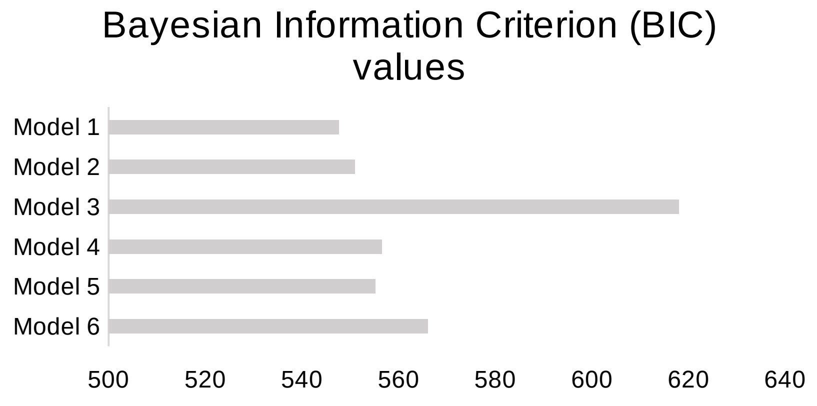

Figure S5. Formal comparison of computational model fit. Bars represent the sum of Bayesian Information Criterion (BIC) values per participant for each model. Smaller BIC values are indicative of a better model fit.

Mood and anxiety measures

To assess effects of tDCS on acute mood and anxiety, we contrasted participants' questionnaire scores completed at the beginning and end of each session. Raw scores on each of the questionnaires are reported in Table 1-1. Analyses were carried out using repeated-measures ANOVAs, with PANAS Positive scores, PANAS Negative scores, and STAI-State scores as dependent variables. tDCS and Time (before vs. after tDCS/task) were included as predictor variables. An interaction between tDCS and Time would be indicative of an effect of tDCS on acute mood or anxiety. We found no such interactions for PANAS Positive scores ($F_{(1,19)} \leq 3.68$, $p \geq 0.072$) or STAI-State scores ($F_{(1,19)}$) ≤ 2.94, $p \geq 0.103$). There was a significant effect of tDCS on PANAS Negative scores with offline prefrontal tDCS (tDCS x Time interaction: $F_{(1,19)} = 5.72$, $p = 0.027$, $\eta^2_G = 0.014$). PANAS Negative scores decreased significantly with sham ($t_{(19)} = 3.39$, $p = 0.003$) but not active tDCS ($t_{(19)} = 0.38$, $p = 0.705$).

Table S2. Mean (SD) PANAS and STAI-state scores by tDCS condition

	Study 1		Study 2		Study 3		Study 4	
	Online DLPFC		*Offline DLPFC*		*Online M1*		*Replication*	
	(N = 20)		*(N = 20)*		*(N = 20)*		*(N = 20)*	
PANAS Positive								
Sham tDCS	26.8	22.4	28.2	26.0	32.5	30.4	31.0	27.4
	(4.9)	(6.2)	(6.4)	(6.4)	(7.3)	(8.7)	(7.10)	(7.3)
Active tDCS	27.2	24.4	28.6	24.4	31.3	28.1	30.6	27.7
	(5.3)	(6.6)	(6.3)	(6.0)	(7.9)	(8.6)	(7.0)	(8.6)
PANAS Negative								
Sham tDCS	10.9	10.4	13.0	11.6	11.0	11.0	11.5	11.4
	(1.1)	(0.6)	(3.0)	(2.6)	(1.9)	(1.4)	(2.0)	(2.4)
Active tDCS	11.6	10.6	12.0	11.9	11.9	10.8	11.8	11.1
	(2.3)	(0.9)	(2.7)	(3.0)	(4.6)	(1.4)	(2.1)	(1.5)
STAI-State								
Sham tDCS	30.0	31.8	29.1	30.0	29.0	29.8	32.2	30.6
	(4.7)	(6.9)	(6.7)	(7.7)	(7.3)	(6.5)	(8.7)	(8.1)
Active tDCS	30.0	31.9	30.5	29.3	30.6	31.8	29.6	30.8
	(6.2)	(4.8)	(7.9)	(6.7)	(7.9)	(6.9)	(7.4)	(8.6)

BDI-II; Beck's Depression Inventory II, PANAS; Positive and Negative Affect Scale; STAI, State-Trait Anxiety Inventory.

Stability of prefrontal tDCS effects over time

The increase in reward learning rates in Win- and Loss-volatile blocks with online prefrontal tDCS was stable over time (tDCS x Outcome x Time interaction: $F_{(1,18)} = 0.56$, $p = 0.464$), although the learning rate difference between sham and active tDCS was numerically greater in blocks 4-5 (after stimulation; see Figure S5).

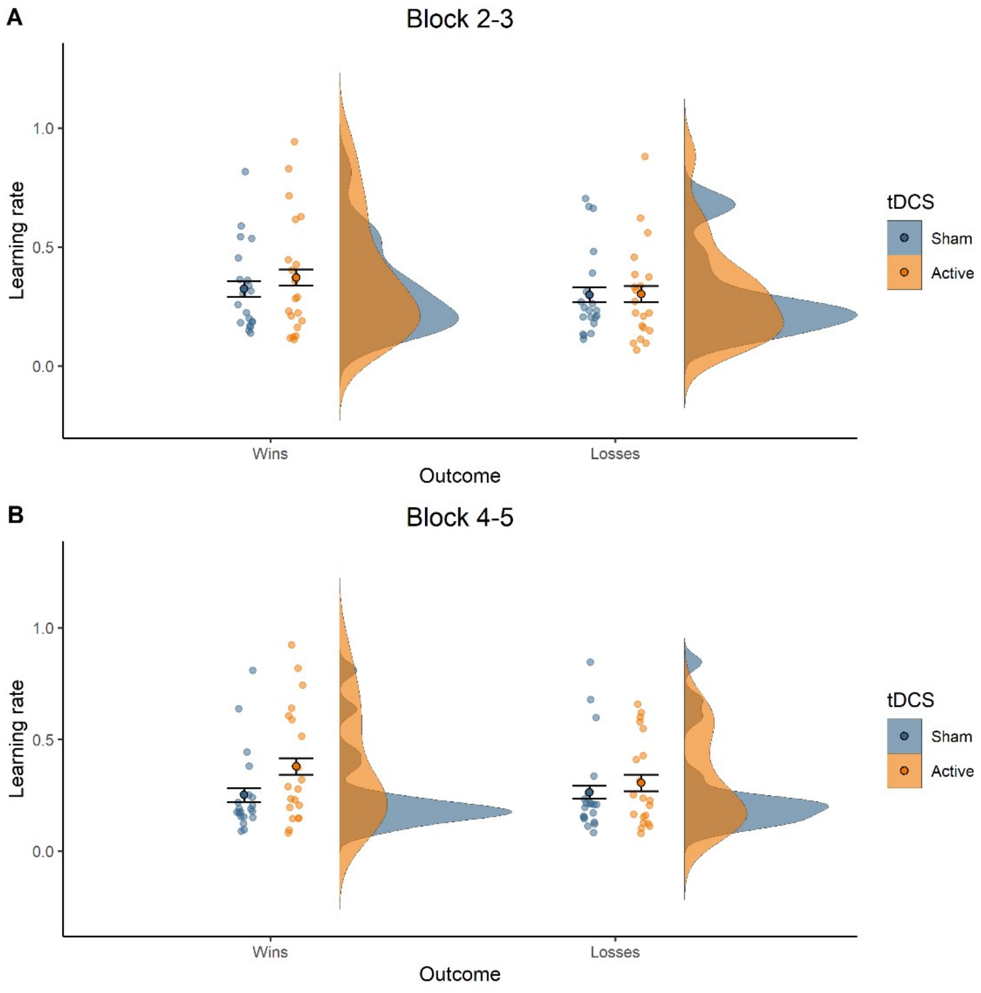

Figure S6. Stable increase in reward learning rates with online prefrontal tDCS. (A) Learning rates in Blocks 2-3 (during tDCS). **(B)** Learning rates in Blocks 4-5 (after tDCS). Violin-plots show the

distribution of learning rates by tDCS condition (sham = blue, active = orange). Summary statistics are provided in boxplots, with the black horizontal line indicating the median and whiskers representing the 25th and 75th percentiles of values. Dots represent participants' individual data points averaged across task blocks.

Table S3. Mean and standard deviation for all model parameters for studies 1-4.

Sample	tDCS Condition	Volatility	Time	Win LR mean	Win LR SD	Loss LR mean	Loss LR SD	Beta mean	Beta SD	Bias term *t* mean	Bias term *t* SD
Bifrontal online	Real	losses-volatile	First half	-0.92	2.01	-0.48	1.07	0.75	0.72	0.02	0.17
Bifrontal online	Real	losses-volatile	Second half	-1.10	1.97	-0.42	1.27	0.79	0.62	0.06	0.16
Bifrontal online	Real	wins-volatile	First half	-0.51	1.10	-1.84	1.53	0.74	0.60	-0.05	0.14
Bifrontal online	Real	wins-volatile	Second half	-0.21	1.36	-1.86	1.54	0.81	0.48	0.01	0.17
Bifrontal online	Real	both-volatile	First half	0.14	1.17	-0.82	1.36	0.84	0.45	0.00	0.13
Bifrontal online	Real	both-volatile	Second half	-0.49	1.40	-1.37	1.40	0.90	0.53	-0.05	0.12
Bifrontal online	Sham	losses-volatile	First half	-1.69	1.18	-0.64	0.97	0.88	0.64	0.02	0.09
Bifrontal online	Sham	losses-volatile	Second half	-2.07	1.15	-0.76	0.94	0.93	0.56	0.05	0.10
Bifrontal online	Sham	wins-volatile	First half	-0.17	1.15	-1.57	1.46	0.92	0.48	0.03	0.14
Bifrontal online	Sham	wins-volatile	Second half	-0.66	1.02	-1.92	1.50	0.94	0.54	-0.09	0.13
Bifrontal online	Sham	both-volatile	First half	-0.36	1.38	-0.91	1.36	0.93	0.39	-0.03	0.15
Bifrontal online	Sham	both-volatile	Second half	-0.95	1.38	-1.06	1.21	1.02	0.47	0.06	0.14
Bifrontal offline	Real	losses-volatile	First half	-1.69	1.44	-1.07	0.87	0.99	0.64	0.01	0.12
Bifrontal offline	Real	losses-volatile	Second half	-1.62	1.45	-0.98	1.25	1.05	0.64	0.00	0.12
Bifrontal offline	Real	wins-volatile	First half	-0.64	0.86	-1.72	1.28	1.18	0.38	0.00	0.11
Bifrontal offline	Real	wins-volatile	Second half	-0.79	0.85	-2.17	1.04	1.01	0.46	-0.01	0.09
Bifrontal offline	Real	both-volatile	First half	-0.61	1.01	-0.65	1.15	1.11	0.32	-0.01	0.16
Bifrontal offline	Real	both-volatile	Second half	-0.92	0.98	-1.55	1.66	1.11	0.41	0.04	0.13
Bifrontal offline	Sham	losses-volatile	First half	-1.21	1.27	-0.67	0.64	0.81	0.72	0.03	0.21
Bifrontal offline	Sham	losses-volatile	Second half	-1.07	1.35	-0.67	0.84	0.88	0.56	0.00	0.27
Bifrontal offline	Sham	wins-volatile	First half	-0.34	1.00	-1.97	1.43	0.94	0.55	-0.03	0.19
Bifrontal offline	Sham	wins-volatile	Second half	-0.50	1.06	-1.42	1.39	0.77	0.70	-0.03	0.18
Bifrontal offline	Sham	both-volatile	First half	-0.33	1.56	-0.21	1.30	0.87	0.59	0.04	0.12
Bifrontal offline	Sham	both-volatile	Second half	-0.79	1.40	-0.87	1.63	0.94	0.65	0.04	0.15

Motor cortex online	Real	losses-volatile	First half	-1.61	1.04	-0.83	0.70	0.95	0.39	0.07	0.14
Motor cortex online	Real	losses-volatile	Second half	-1.62	1.21	-0.95	1.05	1.02	0.41	0.02	0.14
Motor cortex online	Real	wins-volatile	First half	-0.55	0.77	-1.60	1.48	1.03	0.49	0.01	0.11
Motor cortex online	Real	wins-volatile	Second half	-0.57	0.95	-1.93	1.41	1.05	0.60	-0.02	0.12
Motor cortex online	Real	both-volatile	First half	0.09	1.19	-1.28	1.31	0.98	0.43	-0.03	0.08
Motor cortex online	Real	both-volatile	Second half	-0.47	1.26	-1.29	1.85	1.08	0.46	-0.03	0.17
Motor cortex online	Sham	losses-volatile	First half	-1.11	1.91	-0.80	0.88	0.76	0.63	0.10	0.15
Motor cortex online	Sham	losses-volatile	Second half	-1.65	1.42	-1.16	0.70	0.91	0.73	-0.02	0.17
Motor cortex online	Sham	wins-volatile	First half	-0.73	1.05	-2.03	1.17	1.05	0.58	-0.03	0.11
Motor cortex online	Sham	wins-volatile	Second half	-1.03	1.06	-2.54	1.03	1.08	0.43	0.00	0.12
Motor cortex online	Sham	both-volatile	First half	-0.51	1.75	-0.96	1.59	1.05	0.42	-0.01	0.08
Motor cortex online	Sham	both-volatile	Second half	-0.32	1.09	-1.09	1.23	1.01	0.48	0.00	0.17
Replication bifrontal online	Real	losses-volatile	First half	-1.42	1.62	-0.95	0.86	0.97	0.48	0.05	0.16
Replication bifrontal online	Real	losses-volatile	Second half	-1.87	1.57	-0.84	0.51	1.02	0.45	0.04	0.12
Replication bifrontal online	Real	wins-volatile	First half	-0.46	1.14	-1.97	0.87	0.96	0.53	-0.07	0.10
Replication bifrontal online	Real	wins-volatile	Second half	-0.79	1.27	-2.56	1.03	1.17	0.35	-0.02	0.09
Replication bifrontal online	Real	both-volatile	First half	-0.45	1.44	-2.14	1.50	1.17	0.36	-0.03	0.10
Replication bifrontal online	Real	both-volatile	Second half	-1.26	1.23	-1.79	1.08	1.21	0.39	-0.02	0.10
Replication bifrontal online	Sham	losses-volatile	First half	-1.96	0.91	-0.90	0.93	0.91	0.53	0.04	0.10
Replication bifrontal online	Sham	losses-volatile	Second half	-1.98	1.13	-0.85	1.02	0.99	0.59	0.07	0.14
Replication bifrontal online	Sham	wins-volatile	First half	-0.77	1.22	-2.38	1.19	1.04	0.45	0.01	0.18
Replication bifrontal online	Sham	wins-volatile	Second half	-0.80	1.09	-2.36	0.88	1.08	0.35	-0.02	0.08
Replication bifrontal online	Sham	both-volatile	First half	-0.49	1.64	-0.78	1.27	1.02	0.36	-0.01	0.13
Replication bifrontal online	Sham	both-volatile	Second half	-1.04	1.34	-1.80	1.14	1.08	0.51	0.03	0.09

Note that an inverse logistic transform has been applied to the learning rate estimates (LR), and a log transform to the inverse temperature estimates (Beta).

Analysis of the effect of tDCS on inverse temperature and bias term

To test whether online bifrontal tDCS had an effect on the inverse temperature, an ANOVA was run including tDCS, Volatility and Time as within-subject factors and inverse temperature as dependent variable (Study 1). There was no main effect of tDCS (F(1,18) = 2.38, p = .13) or interaction between tDCS and Time (F(1,18) = 0.02, p = .87) or tDCS and Volatility (F(1,18) = 0.01, p = .89). To test whether online bifrontal tDCS had an effect on the bias term t, an ANOVA was run including tDCS, Volatility and Time as within-subject factors and bias term as dependent variable. There was no main effect of tDCS (F(1,18) = 0.30, p = .58) or tDCS and Volatility (F(1,18) = 0.02, p = .88). Although there was a trend towards an interaction effect between tDCS and Time (F(1,18) = 3.96, p = .06), post-hoc paired t-tests indicated that there was no significant effect of tDCS on the bias term during tDCS (F(1,18) = 3.16, p = .09) or after tDCS (F(1,18) = 2.99, p = .10)). The effect of online bifrontal tDCS was therefore specific to the win learning rate.

To test whether *offline* bifrontal tDCS had an effect on the inverse temperature, an ANOVA was run including tDCS, Volatility and Time as within-subject factors and inverse temperature as dependent variable (Study 2). There was no main effect of tDCS (F(1,18) = 3.17, p = .09) or interaction between tDCS and Time (F(1,18) = 0.003, p = .95) or tDCS Volatility (F(1,18) = 0.25, p = .62). To test whether offline bifrontal tDCS had an effect on the bias term t, an ANOVA was run including tDCS, Volatility and Time as within-subject factors and bias term as dependent variable. There was no main effect of tDCS (F(1,18) = 0.05, p = .82), interaction between tDCS and Time (F(1.18 = 0.10, p= . 74), or tDCS and Volatility (F(1,18) = 0.29, p = .59).

To test whether tDCS applied to the motor cortex had an effect on the inverse temperature, an ANOVA was run including tDCS, Volatility and Time as within-subject factors and inverse

temperature as dependent variable (Study 3). There was no main effect of tDCS ($F(1,18) = 0.32$, $p = .57$) or interaction between tDCS and Time ($F(1,18) = 0.19$, $p = .66$). Although there was a trend towards an interaction effect between tDCS and Volatility, post-hoc paired t-tests indicated that there was no significant main effect of tDCS on the inverse temperature in the wins-volatile ($F(1,18) = 0.08$, $p = .77$). or losses-volatile condition ($F(1,18) = 1.24$, $p = .27$). To test whether tDCS applied to the motor cortex had an effect on the bias term *t*, an ANOVA was run including tDCS, Volatility and Time as within-subject factors and bias term as dependent variable. There was no main effect of tDCS ($F(1,18) = 0.22$, $p = .63$), interaction between tDCS and Time (F(1.18 = 0.01, p= . 91), or tDCS and Volatility ($F(1,18) = 0.02$, $p = .87$).

To test whether online bifrontal tDCS had an effect on the inverse temperature in the replication sample, an ANOVA was run including tDCS, Volatility and Time as within-subject factors and inverse temperature as dependent variable in the replication dataset (Study 4). There was no main effect of tDCS ($F(1,18) = 0.14$, $p = .70$) or interaction between tDCS and Time ($F(1,18) = 0.61$, $p = .44$) or tDCS and Volatility ($F(1,18) = 0.09$, $p = .76$). To test whether online bifrontal tDCS had an effect on the bias term *t* in the replication sample, an ANOVA was run including tDCS, Volatility and Time as within-subject factors and bias term as dependent variable. There was no main effect of tDCS ($F(1,18) =3.01$, $p = .09$) or interaction between tDCS and Time ($F(1,18) = 0.24$, $p = .62$) or tDCS and Volatility ($F(1,18) = 0.59$, $p = .44$). Online bifrontal tDCS had therefore no significant effect on any other computational parameter apart from the win learning rate.

Sensitivity power analysis

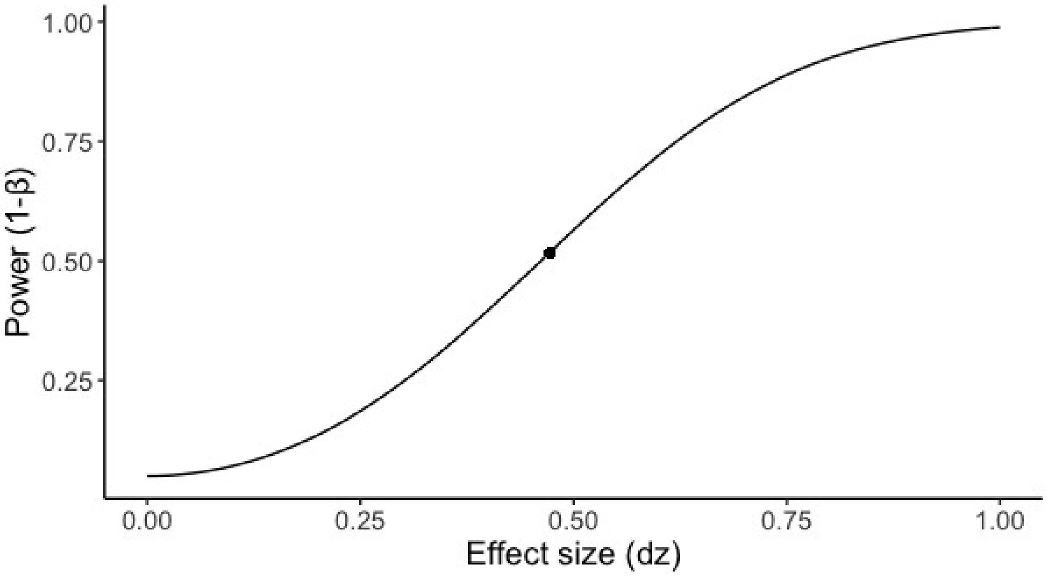

Figure S7. Sensitivity power analysis for a paired t-test with a sample size of N = 20. The dot represents the effect size observed in Study 1 (*dz* = 0.472) and the resulting power (1-β = 0.51).
